# 72. A retrospective diagnosis of Blau syndrome

**DOI:** 10.1093/rap/rky034.035

**Published:** 2018-09-20

**Authors:** Jessica Ellis, Ben Faber, Sarah Emerson

**Affiliations:** Rheumatology Department, Southmead Hospital, Bristol, UNITED KINGDOM

**Introduction:** A 31 female diagnosed with systemic juvenile idiopathic arthritis (SJIA) and uveitis has been managed by our adult rheumatology service since 2007. She has suffered uncontrolled disease activity, with a poor treatment response despite multiple drug regimens. In 2018 her four year old son was diagnosed with Blau syndrome. Given the autosomal dominant inheritance pattern of Blau syndrome, and the presence of atypical features of SJIA, a retrospective review of her initial diagnosis was made.

**Case description:** This patient diagnosed with SJIA and uveitis was transferred under the care of our adult rheumatology service in 2007 at the age of 20, having been first diagnosed at the age of 18 months in 1989. At this time she reported a history of near continuous polyarthritis since diagnosis, with a more recent development of bilateral anterior uveitis in 2003. There was no family history of joint disease. At this time she reported ongoing inflammatory joint symptoms as well as systemic features such as fatigue. Systemic examination revealed small joint synovitis as well as flexion contractures at the elbows. There were no notable skin changes at this time. She has never had any rashes. Eye examination showed the legacy of uncontrolled inflammatory activity with bilateral vitritis and chorioretinal scarring. Repeat investigations at this time showed a negative rheumatoid factor, ANA and ANCA negative alongside a normal complement and raised inflammatory markers. Radiographs of the hands showed symmetrical erosive damage and joint space narrowing, while those of the feet showed only sclerosis and some loss of joint space. Before transferring to adult services multiple medications had been trialled, without achieving successful control of her disease activity. These included naproxen, sodium aurothiomalate, methotrexate, mycophenolate mofetil, etanercept and adalimumab, in addition to near continual use of oral corticosteroids. Utilisation of infliximab had been complicated by anaphylaxis following a second trial of the medication therefore precluding future use. Due to clear evidence of ongoing ocular and joint inflammation in 2007 anakinra was trialled; despite offering initial control she soon relapsed. In an attempt to gain control of her disease activity incremental additions were made to her treatment regimen including rituximab, tacrolimus and mycophenolate mofetil. In 2008 her eye disease was complicated by the development of cataracts. By 2012 she had required bilateral cataract surgery a right surgical iridotomy and a left eye intravitreal dexamethasone implant. Successive trials of alternative biologic therapies have been inadequate to control her joint inflammation. In addition her ocular inflammation remains active, and as such she has required a significant daily dose of oral corticosteroids to achieve disease control. She has suffered progressive erosions with deformity of both large and small joints and in 2017 required a left total hip replacement due to joint degeneration. In 2011, the patient suffered a miscarriage from an unplanned pregnancy whilst on certolizumab. Following this she decided to try for a family and a preconception treatment plan was formed. This involved stopping all disease modifying medication and continuing solely on an increased dose of oral corticosteroid. The patient went on to have two successful pregnancies in 2013 and 2014. Her eldest child remains well, with her youngest child developing of symptoms of an inflammatory polyarthritis aged four years old. He went on to have a genetic testing for a mutation in the NOD2 gene which led to a diagnosis of Blau syndrome. Following her sons diagnosis of Blau syndrome this patient’s original diagnosis of SJIA was revisited. Blau syndrome is an autosomal dominant auto-inflammatory condition characterised by the clinical triad of arthritis, recurrent uveitis and granulomatous skin dermatitis; the absence of symptoms in the son’s father therefore raised the suspicion that our patient had been misdiagnosed. NOD2 is the gene which has been implicated with 22 recorded mutations. NOD2 codes for a receptor involved in bacterial recognition and immune response. Of note, Blau syndrome shares an identical clinical phenotype with early onset sarcoidosis (EOS); these were initially felt to represent different disease entities but the advent of genetic testing has demonstrated that in fact they are the same disease. Some refer to Blau syndrome as the inherited form of the disease and EOS the sporadic form. Given our patient has no family history of inflammatory arthritis it is likely she has a sporadic mutation. Our patient underwent genetic testing, confirming that she had the same mutation as her son and thus supporting a change in her diagnosis. We prefer the terminology of Blau syndrome as an overarching diagnosis for our patient and her son, rather than EOS and Blau respectively, as it clearly communicates the presence of a rare, genetically predetermined, paediatric polyarthritis.

**Discussion:** Like the far more prevalent SJIA, Blau syndrome is a paediatric polyarthritis with systemic manifestations. Despite this overlap there are key differences that have been identified between the two with respect to both clinical findings and treatment strategies. Prevalence of uveitis in patients with Blau syndrome has been estimated to be 81%; this is far rarer in SJIA with only 0.6% of patients affected. Additionally in SJIA the posterior segment involvement, typified by choroidal scars, is never seen whereas it is found in 72% of Blau uveitis. Furthermore, uveitis in Blau syndrome is more likely to be bilateral than JIA and more likely to confer complications such as cataracts and visual impairment. Arthritis in Blau syndrome shows a symmetrical pattern involving both large and small joints. Classically florid, synovitis is exhibited at the joints with tenosynovitis and cysts frequently seen. Flexion deformities in the hands such as campylodactyly are common. Radiologically changes in Blau syndrome classically show no evidence of erosions. This contrasts with our patient whose radiographs have displayed progressive erosive damage; this discrepancy is explained by our patient having a rarer erosive form of Blau syndrome. There is no consensus regarding treatment for patients with Blau syndrome; the evidence suggests that inflammation in Blau syndrome may persist despite combination use of steroids, immunosuppressive drugs and biologics. TNF alpha inhibition and IL-1 blockade, such as those received by our patient, have been seen anecdotally to partially control articular and ocular inflammation respectively. It seems therefore that our patient has been treated appropriately within the current body of evidence that is available, although establishing the diagnosis clearly has importance regarding disease phenotype. Critically had the patients true diagnosis been known, preconception planning with her would have included offering referral for genetic counselling to discuss the implications of having Blau syndrome.

**Key Learning Points:** Blau syndrome is commonly misdiagnosed as SJIA. Consider the alternative diagnosis of Blau syndrome in patients displaying an autosomal dominant inheritance pattern of SJIA. Posterior segment uveitis is not seen in SJIA; its presence should prompt consideration of an alternative diagnosis. Although uncommon, joint erosions on radiology do not preclude a diagnosis of Blau syndrome. Genetic counselling services should be offered to patients with Blau syndrome.

**Disclosure: J. Ellis:** None. **B. Faber:** None. **S. Emerson:** None.

